# Stability of Proton Exchange Membranes in Phosphate Buffer for Enzymatic Fuel Cell Application: Hydration, Conductivity and Mechanical Properties

**DOI:** 10.3390/polym13030475

**Published:** 2021-02-02

**Authors:** Luca Pasquini, Botagoz Zhakisheva, Emanuela Sgreccia, Riccardo Narducci, Maria Luisa Di Vona, Philippe Knauth

**Affiliations:** 1 CNRS, MADIREL (UMR 7246) and International Laboratory: Ionomer Materials for Energy, Aix Marseille Univ, Campus St. Jérôme, 13013 Marseille, France; botagoz.zhakisheva@etu.univ-amu.fr (B.Z.); philippe.knauth@univ-amu.fr (P.K.); 2 Department Industrial Engineering and International Laboratory: Ionomer Materials for Energy, University of Rome Tor Vergata, 00133 Roma, Italy; emanuela.sgreccia@uniroma2.it (E.S.); riccardo.narducci@uniroma2.it (R.N.); divona@uniroma2.it (M.L.D.V.)

**Keywords:** ionomer, blend, casting, SPEEK, SPPSU, crosslinking

## Abstract

Proton-conducting ionomers are widespread materials for application in electrochemical energy storage devices. However, their properties depend strongly on operating conditions. In bio-fuel cells with a separator membrane, the swelling behavior as well as the conductivity need to be optimized with regard to the use of buffer solutions for the stability of the enzyme catalyst. This work presents a study of the hydrolytic stability, conductivity and mechanical behavior of different proton exchange membranes based on sulfonated poly(ether ether ketone) (SPEEK) and sulfonated poly(phenyl sulfone) (SPPSU) ionomers in phosphate buffer solution. The results show that the membrane stability can be adapted by changing the casting solvent (DMSO, water or ethanol) and procedures, including a crosslinking heat treatment, or by blending the two ionomers. A comparison with Nafion^TM^ shows the different behavior of this ionomer versus SPEEK membranes.

## 1. Introduction

Synthetic polymer membranes are economical separator materials in many advanced devices, including ultrafiltration systems, electrolyzers, redox-flow batteries and hydrogen separation [1,2,3,4,5]. Membranes composed of ionomers [6] (i.e., polymers with grafted ionic groups, including commercial Nafion^TM^, 3M^TM^, Aquivion^TM^) are important for the development of highly efficient and reliable electrochemical energy conversion devices such as fuel cells. The main challenge is to increase the ionomer conductivity and maintain an appropriate hydrolytic and mechanical stability [7,8,9]; very often, the good conductivity of highly functionalized polymers is linked to a poor hydrolytic stability [10,11], an increase of the reactant permeability and an overall decay of the device performances, ultimately causing the failure of the systems. The microstructure and behavior of ionomer membranes in operating conditions thus need to be better understood to increase the lifetime and the efficiency of the devices.

In the last years, biological fuel cells (BioFCs) were developed as an alternative to classical fuel cells [12]. The main advancement and, at the same time, the main challenge for this type of cell is the substitution of the inorganic catalyst. Generally, expensive and rare (e.g., platinum) [13,14] BioFCs can be divided in two sub-categories [15], microbial fuel cells (MFCs), using complex organisms as catalyst [16,17], and enzymatic fuel cells (EFCs), using enzymes as catalyst [18,19,20]. In both cases, the coupling of the catalyst with the materials of the whole device has to be deeply understood to guarantee the catalyst’s stability over time and the overall performance to the device [14]. EFCs including a separator membrane [20,21] were introduced to solve the main problems of membrane-less EFC, i.e., the mixing of the reactants (O_2_ and H_2_) and the sensitivity of some enzymes to O_2_ [22], which result in a short lifetime of the device and poor performances [23,24]. In the specific example of the EFCs, the enzyme catalyst is often immersed in a buffer solution to guarantee the optimal activity [24]. All materials utilized are thus in contact with the buffer, including the ionomer membrane that has to be hydrolytically stable and maintain a sufficient ionic conductivity in this medium.

We already demonstrated [25] that the hydrolytic stability and conductivity of proton and anion conducting membranes strongly depends on the type, concentration and pH of the buffer: ionic crosslinks by ions contained in the solution can significantly affect the conductivity. Some previous works [26,27,28,29,30] demonstrate that commercially available Nafion^TM^ membranes can undergo a dramatic proton conductivity reduction in BioFCs related to cation exchange. After a few hours of EFC operation, the membrane completely exchanged cations and required an intensive wash with sulfuric acid or a replacement, increasing the overall maintenance cost of the device. It is thus of crucial importance to study the behavior of ionomers in typical EFC conditions and in particular how the hydrolytic stability, as well as the conductivity, can be optimized by different methods (such as blend formation between polymers, change of the casting procedure and solvent, crosslinking reactions) to boost the overall performances of the device.

**Scheme 1:**
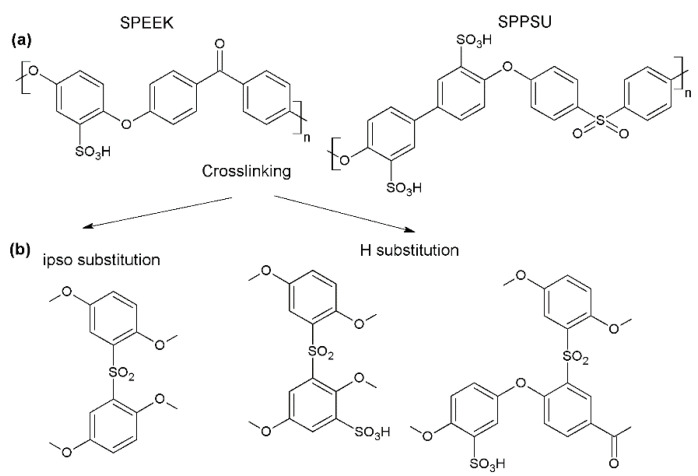
(**a**) Structures of sulfonated poly(ether ether ketone) (SPEEK) and sulfonated poly(phenyl sulfone) (SPPSU). (**b**) Various possible pathways for the reticulation of SPEEK.

In this work, we investigate the casting of two main sulfonated aromatic polymers (SAP), sulfonated poly(ether ether ketone) (SPEEK) and sulfonated poly(phenyl sulfone) (SPPSU), as shown in Scheme 1a, and their blends in different solvents to obtain an optimized membrane for the EFC phosphate buffer solution. In particular, the casting of highly functionalized SPEEK from dimethylsulfoxide (DMSO), DMSO-water, ethanol and ethanol-water, and the blending of SPEEK and SPPSU, are investigated to optimize the conductivity of the membranes while maintaining a good hydrolytic and mechanical stability. The properties of membranes are reported after immersion in a 0.05 M phosphate buffer solution, including conductivity, hydrolytic stability (gravimetric and volumetric solution uptake), dry density and Young’s modulus. These properties are finally compared to commercial Nafion^TM^ 212 that is used as a benchmark.

## 2. Materials and Methods

The Nafion^TM^ 212 membrane was purchased from Fuel Cell Store (College Station, TX, USA) in the form of a film of 30 × 30 cm^2^ dimension.

Sulfonated poly(ether ether ketone) (SPEEK) and sulfonated poly(phenyl sulfone) (SPPSU) were prepared by reaction of poly(ether ether ketone) (Victrex, Thornton-Cleveleys, UK, MW = 38,300 g/mol) or poly(phenyl sulfone) (Solvay, Brussels, Belgium, MW = 46,173 g/mol) with concentrated sulfuric acid (H_2_SO_4_; Aldrich, St. Louis, MO, USA, 95–97%) under nitrogen atmosphere [31].

The degree of sulfonation (DS) and the ion exchange capacity (IEC) of each polymer was determined by NMR spectroscopy and acid-base titration [32,33]. The obtained values were, for SPEEK: DS = 92% and IEC = 2.50 meq/g, and for SPPSU: DS = 152% and IEC = 2.92 meq/g.

**Figure 1:**
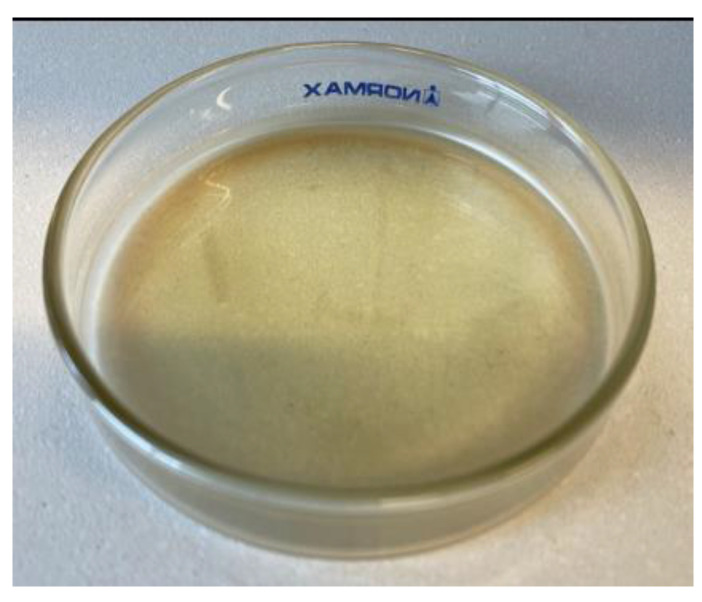
A typical membrane cast in a flat Petri dish.

Membranes were cast from DMSO, DMSO-water, ethanol or ethanol-water in a flat Petri dish (Figure 1). Generally, 0.5 g of polymer (or a mixture of two polymers) was dissolved in 10 g of solvent (or a mixture of two solvents). In the case of DMSO or DMSO-water, after evaporation to around one third of the original volume, the solution was poured in a Petri dish and evaporated in an oven at 80 °C for 18 h.

For ethanol and ethanol-water, the solution was directly poured into a Petri dish and evaporated in an oven at 80 °C for 15 min or 18 h, respectively.

A crosslinking treatment at 180 °C for 3 h, as described in References [11,34,35], was applied to as-cast membranes that showed poor hydrolytic stability (in particular, SPPSU-based membranes).

### 2.1. Hydrolytic Stability

The hydrolytic stability was investigated in a 0.05 M phosphate (H_2_PO_4_^−^/HPO_4_^2−^) buffer at pH = 6.5 ± 0.2. The pH was determined with a calibrated pH-meter (Mettler Toledo). This buffer was chosen because it is among the best buffer solutions that stabilize most of the enzymes in EFCs, in particular bilirubin oxidase [12,36].

Mass uptake (*MU*) was measured twice at 25 °C and calculated according to the equation:

(1) $$MU\left(\%\right)=\frac{m_{wet}-m_{dry}}{m_{dry}}a\times 100$$

The mass of wet samples (*m_wet_*) was determined after immersion in the buffer solution during 24 h at 25 °C in a thermoregulated oven without any additional washing in water. Before the measurement, the membrane was wiped carefully with absorbing paper to remove the excess of buffer solution on the surface. The mass of the dry samples (*m_dry_*) was measured in a closed vessel after drying over P_2_O_5_ for 24 h.

The dry density of the ionomer was measured using the mass and dimensions of the membranes after drying over P_2_O_5_ for 24 h.

### 2.2. Ionic Conductivity

**Figure 2:**
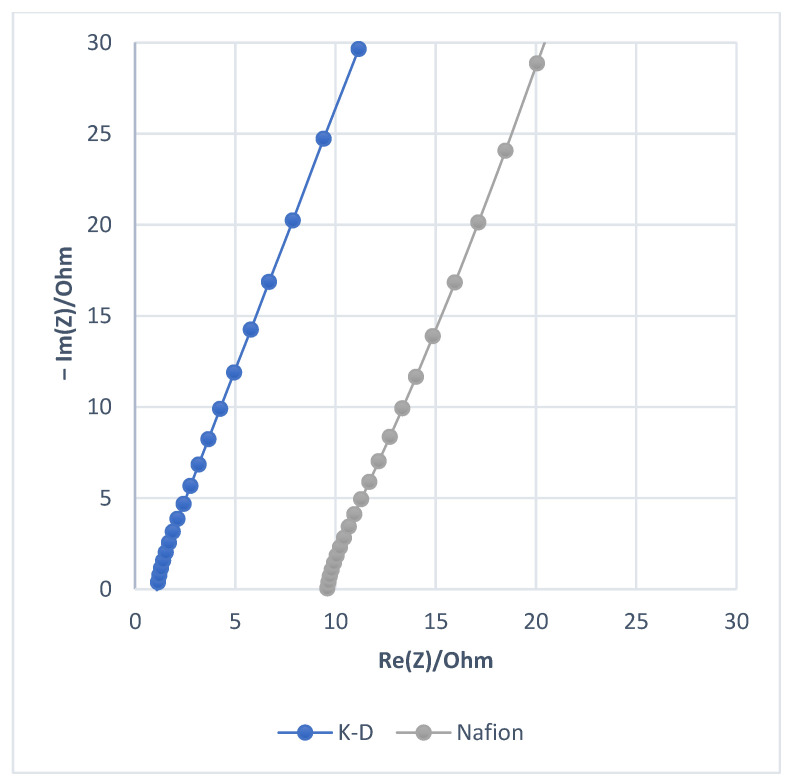
Typical complex impedance diagrams of a SPEEK membrane cast in DMSO (K-D) and a Nafion^TM^ membrane after immersion in buffer.

The through-plane ionic conductivity was measured by impedance spectrometry between 1 Hz and 6 MHz using an impedance spectrometer, Biologic VSP300 (Biologic, Seyssinet-Pariset, France). The amplitude of the oscillating voltage was 20 mV. After immersion in the buffer during 24 h at 25 °C in a thermoregulated oven and after removing the buffer excess on the surface, the samples were measured at 25 °C in humidified conditions inside a Swagelok cell with two stainless-steel electrodes. The sample resistance, R, was obtained from typical impedance spectra (Figure 2) using the intercept with the real axis. The ionic conductivity, *σ,* was calculated using the equation:

(2) $$\sigma =\;\frac{th_{wet}}{R\;a\cdot \;A_{wet}}$$

where *th_wet_* and *A_wet_* are respectively the thickness of the membrane in the wet state after the measurement (measured with a micrometer, Mitutoyo 293-230) and the electrode area.

### 2.3. Tensile Stress-Strain Tests

The mechanical measurements were performed with an Adamel Lhomargy TESTOMETRIC M250-2.5CT testing machine (Testometric, Rochdale, UK). The specimens (two for each test) were cut in rectangular shape (25 mm length and 5 mm width) by paying particular attention to having smooth and perfectly sharp edges to avoid points of stress concentration that eventually lead to premature breaking. The samples were placed between two clamps and the test was performed at a constant elongation rate of 5 mm/min at ambient temperature (25 ± 1 °C) and relative humidity (RH 40% ± 10%), or after immersion at 25 °C in the phosphate buffer solution.

## 3. Results

### 3.1. Cast Membranes

The casting conditions of the realized membranes are reported in Table 1.

The various polymer membranes were realized to study how the casting procedure affected the membrane behavior in the buffer solution. In particular, the utilization of solvents different from DMSO was explored to cast membranes free of harmful solvent for enzymes. The casting temperature of 80 °C was chosen following our previous experience with membranes as energy devices [33,37,38]: it is compatible both with DMSO and ethanol and in the case of ethanol, avoids a too fast evaporation resulting in an inhomogeneous membrane.

SPEEK/SPPSU blends were explored to simultaneously enhance the conductivity and hydrolytic stability of the membrane: SPEEK contributed to the hydrolytic stability and SPPSU contributed to increase the ionic conductivity. Following the results of the characterization of SPEEK/SPPSU blends, we moved from the initial 50/50 wt % to a 70/30 wt % composition, because of the poor stability in the buffer of the first blend (cf. Discussion, Section 4).

The crosslinked membranes (indicated with XL) were realized with the aim of hydrolytic stabilization even with a small loss of ionic conductivity because of the decrease of the ion exchange capacity (IEC) due to the reticulation reaction [35,39]. As described in Scheme 1b, the crosslinking treatment consumes some sulfonic acid groups for the creation of sulfone bridges between chains: following XL, the polymer absorbs less water, because of the loss of some hydrophilic sulfonic acid groups resulting in an increase of the hydrolytic stability. A better compromise between stability and good ionic conductivity is thus possible to reach by reticulation.

The hydrolytic stability and conductivity in phosphate buffer solution, as well as the dry density and Young’s modulus of membranes, are reported in Table 2.

The dry density of membranes depends strongly on the casting solvent. The dry density of SPEEK and SPPSU has the highest value of around 1.4 g/cm^3^ when cast from DMSO, in good agreement with literature values [9,40]. This result indicates a high packing density of the macromolecules, also increasing the stiffness of the membranes by Van der Waals interactions (see below). SPEEK membranes cast from ethanol present a lower density, around 1 g/cm^3^, indicating that in this solvent, the macromolecular chains are much less densely packed with no nanophase separation (cf. Discussion, Section 4). Intermediate values, more similar to DMSO, are observed in ethanol/water mixtures. In the case of SPPSU and SPPSU/SPEEK blends, the dry density is around 1.2 g/cm^3^ and increases as expected after a cross-linking treatment.

### 3.2. MU, Dry Density, Conductivity, Mechanical Properties

Figure 2 shows typical impedance spectra of SPEEK (K-D) and Nafion^TM^ membranes.

### 3.3. Comparison of SPEEK vs. Nafion^TM^

The MU, conductivity and mechanical properties were compared for a typical DMSO-cast SPEEK membrane (K-D) and a Nafion^TM^ 212 membrane before and after immersion in buffer solution.

**Figure 3:**
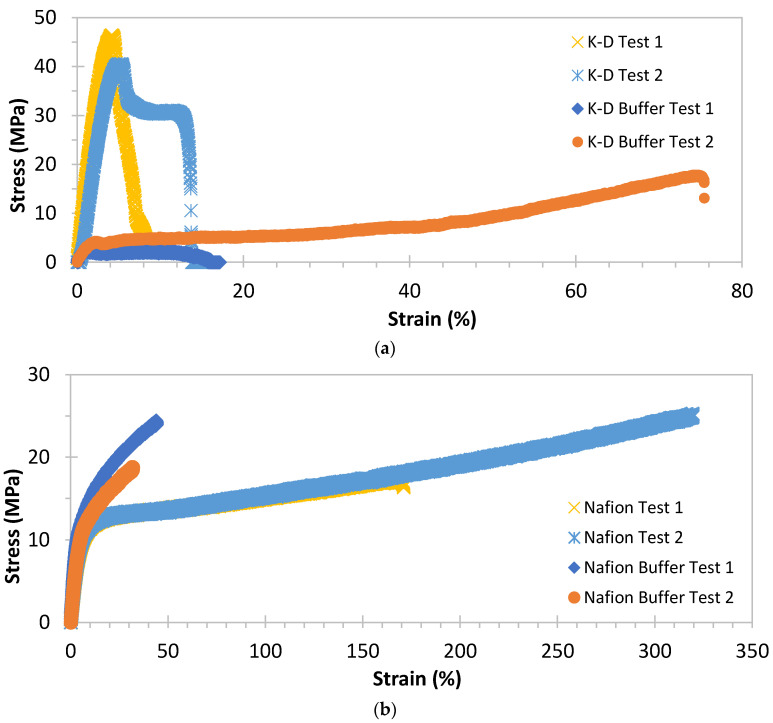
Typical stress-strain curves at relative humidity (RH) = 40% ± 10% and after immersion in buffer solution of (**a**) K-D and (**b**) Nafion 212 membrane.

Figure 3 presents typical stress-strain curves for SPEEK and Nafion^TM^. The mechanical properties are reported in Table 3. The curves and properties in ambient humidity are consistent with previously reported data [40,41]. After immersion in buffer solutions, the curves change dramatically. Whereas the decrease of Young’s modulus and tensile strength of SPEEK are attributable to the plasticizing effect of liquid water due to its high dielectric constant, the behavior of Nafion^TM^ in the buffer is more surprising. One notices a very strong reduction of the elongation at break and a significant increase of Young’s modulus. The enhanced stiffness (and reduced ductility) of Nafion^TM^ is corroborated by the handling experience after buffer immersion.

Analyzing the mechanical test results (Table 3 and Figure 3), the behavior of SPEEK moves from a rigid polymer to a plastic one: The Young’s modulus decreased and the elongation at break increased remarkably. This is certainly due to the plasticizing effect of the water inside the membrane, weakening the interactions between the macromolecular chains and/or functionalized groups [40,42]. In the case of Nafion^TM^, the membrane evolves from a plastic to a rigid behavior after immersion inside the buffer solution. As already demonstrated [41], Nafion^TM^ exchanged with different cations shows a distinct shift to higher temperature of the glass transition, indicating an enhancement of the stiffness. Similar findings were reported in the literature: ion exchange in hydrated Nafion^TM^ samples increases the Young’s modulus of the membranes in increasing order of ionic radius [43]. An increase in Young’s modulus means that the material becomes stiffer and a larger force is necessary to cause elastic deformation.

## 4. Discussion

The difference in mechanical and solubility properties of cast and thermally treated (annealed and commercial) Nafion^TM^ films is usually ascribed to the thermal reorganization of the cold cast micellar structure with sulfonate groups on the outside of the micelle to an inverted micellar structure with the sulfonate groups on the inside [44].

**Figure 4:**
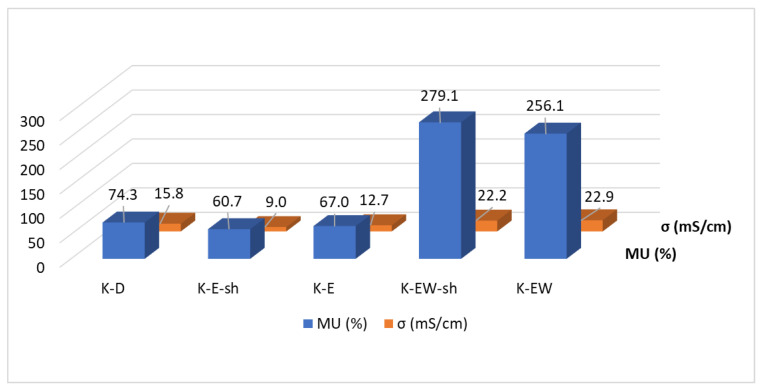
Comparison of SPEEK membranes realized with different solvents and casting procedures.

If we compare membranes cast from 100% SPEEK in different solvents and with different thermal treatment times (Figure 4), we can observe that the thermal treatment time (18 h or 15 min) does not affect the MU and conductivity and that membranes cast from DMSO and ethanol solutions have comparable properties. However, a very large enhancement of solution uptake and conductivity is observed for ethanol-water mixtures. This can be due to the different nanostructure in the membranes related to the high dielectric constant of water: the presence of water allows the formation of ionic clusters between sulfonic acid groups that are responsible for the swelling and the ionic conductivity [45]. The higher conductivity can also be explained by the large ionic mobility in the presence of a large amount of water (see discussion below).

The thermal treatment of 18 h has a clear influence on the elastic modulus, which is much lower in comparison with the samples heated for 15 min only. The initially very stiff membranes deform easier. One can also observe that samples made from pure DMSO show the highest Young’s modulus, and this high stiffness was reported before [11,37,46]. The presence of water in the casting solvent generally further enhances the Young’s modulus. Membranes cast from pure ethanol instead show a quite low stiffness and, in the case of SPEEK, a low density. This indicates a low packing density of the macromolecular chains and low nanophase separation in accordance with the low conductivity.

Comparing membranes realized in 100% DMSO in Table 2, we can observe that the 100% SPPSU membrane dissolves in the buffer solution. The addition of 50% of SPEEK stabilizes the membrane but the apparent solution uptake reveals a partial dissolution phenomenon, attributable to some SPPSU loss in the buffer solution. The two membranes can be further stabilized by a crosslinking treatment. By adding 70% of SPEEK, the membrane does not dissolve, and the conductivity is high due to the effect of a large solution uptake.

**Figure 5:**
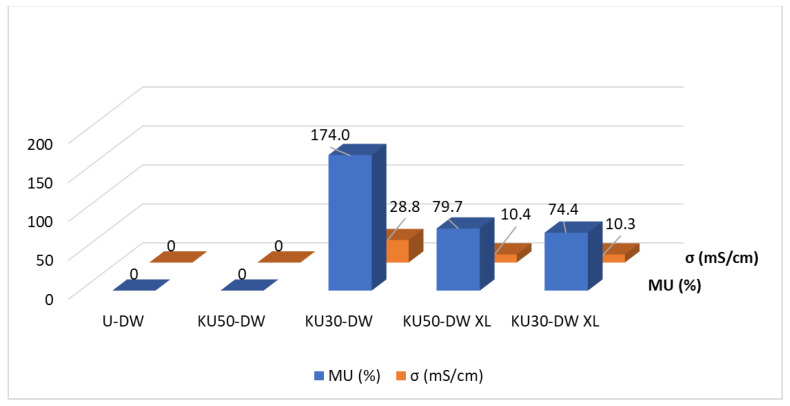
Comparison of SPEEK/SPPSU blend membranes cast from 10% DMSO and 90% water and crosslinked.

Figure 5 shows membranes realized in 10% DMSO and 90% water composed of SPPSU or blended with SPEEK. We can observe, as already mentioned for the SPEEK membranes, that the addition of water to the casting solvent negatively affects the hydrolytic stability of the membranes. The 30/70 membrane (KU30-DW) has a higher MU than a membrane cast in pure DMSO and can be stabilized only with a crosslinking treatment. XL membranes present a good ionic conductivity, and their properties are similar to the reference SPEEK membrane cast from DMSO (K-D).

Membranes with 100% SPPSU cast from ethanol with two different thermal treatment times (U-E-sh and U-E in Table 2) are not stable, and the solvent and the treatment time do not modify the stability. These membranes need to be further crosslinked to achieve the right properties.

**Figure 6:**
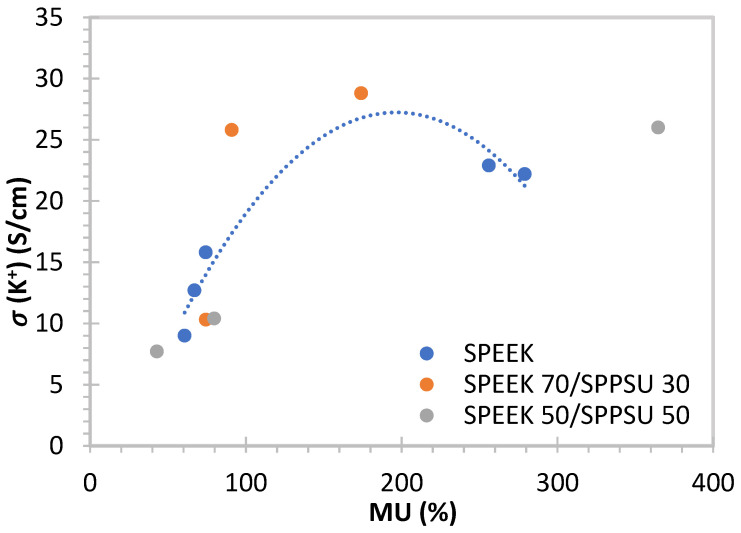
*K*^+^ ion conductivity as a function of the mass uptake. The dotted line is a guide for the eye only.

Figure 6 shows the ionic conductivity as a function of the mass uptake (MU). The apparent maximum at intermediate values of MU was reported before in other ionomers [47]. It is related to antagonistic effects of a cation concentration increase that simultaneously reduces the cation mobility so that an optimal value is obtained for an intermediate value of concentration.

We can further utilize all the data from the mass uptake and conductivity measurement to calculate an effective cation mobility (*K*^+^), assuming that the ion exchange groups have been fully substituted by potassium. Furthermore, we assume that no supplementary adsorbed ions are present, because the exchange was made in a 0.05 M buffer so that the driving force for excess ion adsorption is low. The cation concentration expressed in mol/L in the membrane is then defined by the *IEC* and the *MU*. The density, *ρ*, of the electrolytic solution is taken as 1 g/cm^3^ [48,49].

(3) $$c(K^{+})=\frac{IEC\times \rho }{MU}$$

The effective mobility *u*(*K*^+^) (in cm^2^ V^−1^ s^−1^) is defined according to Equation (4) by the measured ionic conductivity *σ*(*K*^+^) (expressed in mS/cm, Table 2) and the calculated ion concentration *c*(*K*^+^) (in mol/L):

(4) $$u\left(K^{+}\right)=\;\frac{\sigma \left(K^{+}\right)}{F\;a\cdot c\left(K^{+}\right)}$$

**Figure 7:**
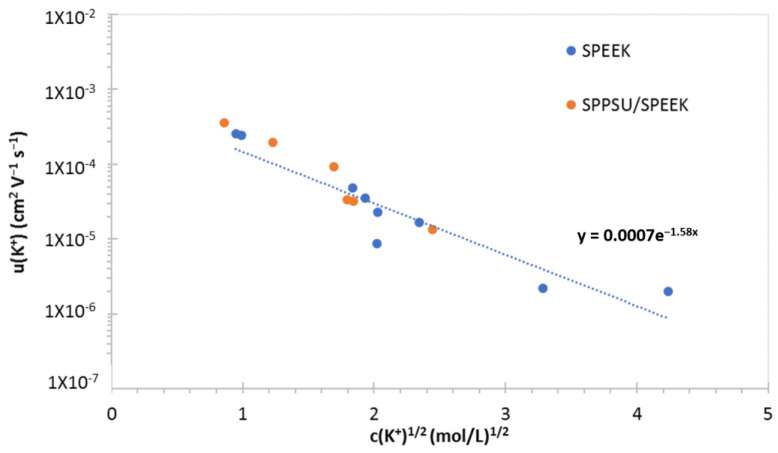
*K*^+^ ion mobility in various proton exchange membranes based on SPEEK and SPPSU/SPEEK blends.

The calculated cation mobility data are reported in Figure 7.

The exponential dependence of the cation mobility as a function of the square root of cation concentration has been previously related to the conditions of ionic motion in nanometric ion conduction channels, where the mobile ions migrate with immobile counterions grafted on the channel walls [50,51,52]. The extrapolation to *c*(*i*) = 0 gives the cation mobility at infinite dilution (*u*(*K*^+^) = 7 × 10^−4^ cm^2^ V^−1^ s^−1^) that can be compared with literature data of *K*^+^ mobility in aqueous solution (*u*(*K*^+^) = 7.6 × 10^−4^ cm^2^ V^−1^ s^−1^) [53,54].

## 5. Conclusions

This work explored the possibility to adapt the hydrolytic behavior, the conductivity and the mechanical properties of ion-conducting membranes based on sulfonated aromatic polymers such as SPEEK and SPPSU by changing the casting solvent and procedure, by blending two different polymers and by a crosslinking treatment. The results of the different characterizations were discussed to find out the optimal compromise for the utilization of these polymers as membranes in EFC containing a buffer solution. The solvent and the casting procedure influenced the properties of the membranes: the use of ethanol reduced the swelling, but at the same time also the Young’s modulus. A shorter casting time resulted in stiffer membranes but had no effect on MU and conductivity. The crosslinking stabilized the membranes that showed a better hydrolytic stability, even if with a little decrease of conductivity. The addition of SPPSU to SPEEK membranes improved the ionic conductivity; however, the ratio between the two polymers has to be carefully tuned in order to keep the hydrolytic stability.

The comparison between a DMSO-cast SPEEK membrane and Nafion^TM^ shows how the behavior of the two polymers before and after the immersion in the buffer solution is different. The SPEEK membrane exhibited a higher MU and conductivity in comparison to Nafion^TM^, and this difference can be ascribed to the solution uptake and the quite high cation mobility in this SAP. The mechanical properties change differently in the two polymers: SPEEK moves from a rigid to a plastic behavior while Nafion^TM^ does exactly the contrary.

The analysis of all data demonstrated that an optimal value of conductivity can be obtained at intermediate values of mass uptake and that the extrapolation to infinite dilution of the mobility of conducting potassium ions is in good agreement with literature data.

This study opens interesting perspectives for the utilization of SAP polymers as membranes in enzymatic and Bio-FC, exploring the possibility of less hazardous casting solvents, while maintaining appropriate conductivity and hydrolytic stability of membranes in buffer solutions.

## Figures and Tables

**Table 1:** Casting conditions of membranes. K stands for SPEEK, U for SPPSU, D for DMSO, W for water, and E for ethanol. XL (crosslink) indicates membranes thermally treated 3 h at 180 °C. sh specifies a short (15 min) heat treatment during casting. The casting temperature in all cases is 80 °C.

Name	Polymer (wt %)		Casting Solvent (wt %)			Casting Time
	SPPSU	SPEEK	DMSO	Water	Ethanol	
K-D	-	100	100	-	-	18 h
K-E-sh	-	100	-	-	100	15 min
K-E	-	100	-	-	100	18 h
K-EW-sh	-	100	-	50	50	15 min
K-EW	-	100	-	50	50	18 h
U-D	100	-	100	-	-	18 h
KU50-D	50	50	100	-	-	18 h
KU30-D	30	70	100	-	-	18 h
U-DW	100	-	10	90	-	18 h
KU50-DW (XL)	50	50	10	90	-	18 h
KU30-DW (XL)	30	70	10	90	-	18 h
U-E-sh	100	-	-	-	100	15 min
U-E	100	-	-	-	100	18 h

**Table 2:** Mass (MU) and volume (VU) uptake, conductivity (σ), dry density (d) and Young’s modulus (E) in humid air of various ionomer membranes. K stands for SPEEK, U for SPPSU, D for DMSO, W for water, and E for ethanol. XL (crosslink) indicates membranes thermally treated for 3 h at 180 °C. sh specifies a short (15 min) heat treatment during casting.

Samples	MU (%)	VU (%)	σ (mS/cm)	d (g/cm^3^)	E (MPa)
K-D	74.3	115.9	15.8	1.44	1400 ± 150
K-E-sh	60.7	98.8	9.0	1.09	900 ± 160
K-E	67.0	110.9	12.7	0.99	580 ± 10
K-EW-sh	279.1	419.7	22.2	1.31	1370 ± 10
K-EW	256.1	382.8	22.9	1.39	1090 ± 5
U-D	Diss *	Diss *	Diss *	1.32	700 ± 50
KU50-D	partial diss	not measured	25.9	1.19	370 ± 80
KU30-D	91	176.4	25.8	1.06	640 ± 10
U-DW	Diss *	Diss *	Diss *	1.15	590 ± 280
KU50-DW	Diss *	Diss *	Diss *	1.13	1220 ± 10
KU30-DW	174	214.4	28.8	1.15	830 ± 120
KU50-DW XL	79.7	192.0	10.4	1.61	not measured
KU30-DW XL	74.4	80.8	10.3	1.27	not measured
U-E-sh	Diss *	Diss *	Diss *	1.23	830 ± 120
U-E	Diss *	Diss *	Diss *	1.20	500 ± 40

* Dissolution.

**Table 3:** Mass uptake (MU), ionic conductivity, σ, and mechanical properties after conditioning in humid air or buffer solution at room temperature (RT). * in fully hydrated conditions.

Membrane	Conditions	MU (%)	σ (mS/cm)	Young’s Modulus (MPa)	Yield Stress (MPa)	Ultimate Strength (MPa)	Elongation at Break (%)
Nafion^TM^ 212	RH = 40% ± 10%	19.0	15.4 *	203 ± 26	5 ± 1	21 ± 6	245 ± 105
	Buffer	9.0	1.9	352 ± 88	5 ± 2	22 ± 4	38 ± 4
K-D	RH = 40% ± 10%	diss	diss	1423 ± 209	25 ± 8	44 ± 4	9 ± 6
	Buffer	74.3	15.8	295 ± 55	3 ± 2	10 ± 8	45 ± 30

## Data Availability

Not applicable.

## References

[1] Zhang W., Shi Z., Zhang F., Liu X., Jin J., Jiang L. (2013). Superhydrophobic and superoleophilic pvdf membranes for effective separation of water-in-oil emulsions with high flux. Adv. Mater..

[2] Skulimowska A., Dupont M., Zatoń M., Sunde S., Merlo L., Jones D., Rozière J. (2014). Proton exchange membrane water electrolysis with short-side-chain Aquivion^®^ membrane and IrO_2_ anode catalyst. Int. J. Hydrog. Energy.

[3] Marini S., Salvi P., Nelli P., Pesenti R., Villa M., Berrettoni M., Zangari G., Kiros Y. (2012). Advanced alkaline water electrolysis. Electrochim. Acta.

[4] Doan T.N.L., Hoang T.K.A., Chen P. (2015). Recent development of polymer membranes as separators for all-vanadium redox flow batteries. RSC Adv..

[5] Kreuer K.D. (2014). Ion conducting membranes for fuel cells and other electrochemical devices. Chem. Mater..

[6] Hess M., Jones R.G., Kahovec J., Kitayama T., Kratochvíl P., Kubisa P., Mormann W., Stepto R.F.T., Tabak D., Vohlídal J. (2006). Terminology of polymers containing ionizable or ionic groups and of polymers containing ions (IUPAC Recommendations 2006). Pure Appl. Chem..

[7] Zahn R., Vörös J., Zambelli T. (2010). Swelling of electrochemically active polyelectrolyte multilayers. Curr. Opin. Colloid Interface Sci..

[8] Kreuer K.-D. (2013). The role of internal pressure for the hydration and transport properties of ionomers and polyelectrolytes. Solid State Ion..

[9] Knauth P., Sgreccia E., Di Vona M. (2014). Chemomechanics of acidic ionomers: Hydration isotherms and physical model. J. Power Sources.

[10] Kim J.-D., Donnadio A., Jun M.-S., Di Vona M.L. (2013). Crosslinked SPES-SPPSU membranes for high temperature PEMFCs. Int. J. Hydrog. Energy.

[11] Hou H., Di Vona M.L., Knauth P. (2012). Building bridges: Crosslinking of sulfonated aromatic polymers—A review. J. Membr. Sci..

[12] De Poulpiquet A., Ranava D., Monsalve K., Giudici-Orticoni M.-T., Lojou E. (2014). Biohydrogen for a new generation of H_2_/O_2_ Biofuel cells: A sustainable energy perspective. Chemelectrochem.

[13] Mazurenko I., Monsalve K., Infossi P., Giudici-Orticoni M.-T., Topin F., Mano N., Lojou E. (2017). Impact of substrate diffusion and enzyme distribution in 3D-porous electrodes: A combined electrochemical and modelling study of a thermostable H_2_/O_2_ enzymatic fuel cell. Energy Environ. Sci..

[14] Bullen R.A., Arnot T.C., Lakeman J.B., Walsh F.C. (2006). Biofuel cells and their development. Biosens. Bioelectron..

[15] Squadrito G., Cristiani P., Subramani V., Bsile A., Verziroglu N. (2016). Microbial and enzymatic fuel cells. Compendium of Hydrogen Energy.

[16] Rosenbaum M.A., Aulenta F., Villano M., Angenent L.T. (2011). Cathodes as electron donors for microbial metabolism: Which extracellular electron transfer mechanisms are involved?. Bioresour. Technol..

[17] Cristiani P., Franzetti A., Gandolfi I., Guerrini E., Bestetti G. (2013). Bacterial DGGE fingerprints of biofilms on electrodes of membraneless microbial fuel cells. Int. Biodeterior. Biodegrad..

[18] Yahiro A.T., Lee S., Kimble D.O. (1964). Bioelectrochemistry. Biochim. Biophys. Acta Sect. Biophys. Subj..

[19] Barton S.C., Gallaway J., Atanassov P. (2004). Enzymatic biofuel cells for implantable and microscale devices. Chem. Rev..

[20] Naidoo S., Naidoo Q., Blottnitz H., Vaivars G. (2013). Glucose oxidase as a biocatalytic enzyme-based bio-fuel cell using Nafion membrane limiting crossover. IOP Conf. Ser. Mater. Sci. Eng..

[21] Moehlenbrock M.J., Minteer S.D. (2008). Extended lifetime biofuel cells. Chem. Soc. Rev..

[22] Lalaoui N., De Poulpiquet A., Haddad R., Le Goff A., Holzinger M., Gounel S., Mermoux M., Infossi P., Mano N., Lojou E. (2015). A membraneless air-breathing hydrogen biofuel cell based on direct wiring of thermostable enzymes on carbon nanotube electrodes. Chem. Commun..

[23] Mazurenko I., Wang X., De Poulpiquet A., Lojou E. (2017). H2/O2enzymatic fuel cells: From proof-of-concept to powerful devices. Sustain. Energy Fuels.

[24] Xiao X., Xia H.-Q., Wu R., Bai L., Yan L., Magner E., Cosnier S., Lojou E., Zhu Z., Liu A. (2019). tackling the challenges of enzymatic (bio)fuel cells. Chem. Rev..

[25] Pasquini L., Wacrenier O., Di Vona M., Knauth P. (2018). Hydration and ionic conductivity of model cation and anion-conducting ionomers in buffer solutions (Phosphate, Acetate, Citrate). J. Phys. Chem. B.

[26] Choi M.-J., Chae K.-J., Ajayi F.F., Kim K.-Y., Yu H.-W., won Kim C., Kim I.S. (2011). Effects of biofouling on ion transport through cation exchange membranes and microbial fuel cell performance. Bioresour. Technol..

[27] Xu J., Sheng G.-P., Luo H.-W., Li W.-W., Wang L.-F., Yu H.-Q. (2012). Fouling of proton exchange membrane (PEM) deteriorates the performance of microbial fuel cell. Water Res..

[28] Rozendal R.A., Hamelers H.V.M., Buisman C.J.N. (2006). Effects of membrane cation transport on pH and microbial fuel cell performance. Environ. Sci. Technol..

[29] Bahar T., Yazici M.S. (2019). Performance assessment of a perfluorosulfonic acid-type membrane (i.e., Nafion™ 115) for an enzymatic fuel cell. Electroanalysis.

[30] Rozendal R.A., Hamelers H.V., Molenkamp R.J., Buisman C.J.N. (2007). Performance of single chamber biocatalyzed electrolysis with different types of ion exchange membranes. Water Res..

[31] Di Vona M.L., Luchetti L., Spera G.P., Sgreccia E., Knauth P. (2008). Synthetic strategies for the preparation of proton-conducting hybrid polymers based on PEEK and PPSU for PEM fuel cells. Comptes Rendus Chim..

[32] Zaidi S.M.J., Mikhailenko S.D., Robertson G., Guiver M., Kaliaguine S. (2000). Proton conducting composite membranes from polyether ether ketone and heteropolyacids for fuel cell applications. J. Membr. Sci..

[33] Di Vona M.L., Sgreccia E., Licoccia S., Alberti G., Tortet L., Knauth P. (2009). Analysis of temperature-promoted and solvent-assisted cross-linking in sulfonated poly(ether ether ketone) (SPEEK) proton-conducting membranes. J. Phys. Chem. B.

[34] Di Vona M.L., Alberti G., Sgreccia E., Casciola M., Knauth P. (2012). High performance sulfonated aromatic ionomers by solvothermal macromolecular synthesis. Int. J. Hydrog. Energy.

[35] Maranesi B., Hou H., Polini R., Sgreccia E., Alberti G., Narducci R., Knauth P., Di Vona M.L. (2013). Cross-linking of sulfonated poly(ether ether ketone) by thermal treatment: How does the reaction occur?. Fuel Cells.

[36] Hitaishi V.P., Mazurenko I., Harb M., Clément R., Taris M., Castano S., Duché D., Lecomte S., Ilbert M., De Poulpiquet A. (2018). Electrostatic-driven activity, loading, dynamics, and stability of a redox enzyme on functionalized-gold electrodes for bioelectrocatalysis. ACS Catal..

[37] Barbieri G., Brunetti A., Di Vona M.L., Sgreccia E., Knauth P., Hou H., Hempelmann R., Arena F., Beretta L.D., Bauer B. (2016). LoLiPEM: Long life proton exchange membrane fuel cells. Int. J. Hydrog. Energy.

[38] Knauth P., Pasquini L., Di Vona M.L. (2017). Comparative study of the cation permeability of protonic, anionic and ampholytic membranes. Solid State Ion..

[39] Knauth P., Pasquini L., Maranesi B., Pelzer K., Polini R., Di Vona M.L. (2013). Proton mobility in sulfonated polyetheretherketone (speEK): Influence of thermal crosslinking and annealing. Fuel Cells.

[40] Narducci R., Di Vona M.L., Knauth P. (2014). Cation-conducting ionomers made by ion exchange of sulfonated poly-ether-ether-ketone: Hydration, mechanical and thermal properties and ionic conductivity. J. Membr. Sci..

[41] Chailan J.-F., Khadhraoui M., Knauth P., Di Vona M.L., Knauth P. (2012). Mechanical and dynamic mechanical analysis of proton-conducting polymers. Solid State Proton Conductors.

[42] Kim Y.S., Dong L., Hickner M.A., Pivovar B.S., McGrath J.E. (2003). Processing induced morphological development in hydrated sulfonated poly(arylene ether sulfone) copolymer membranes. Polymer.

[43] Kundu S., Simon L.C., Fowler M., Grot S. (2005). Mechanical properties of Nafion™ electrolyte membranes under hydrated conditions. Polymer.

[44] Zook L.A., Leddy J. (1996). Density and solubility of nafion: Recast, annealed, and commercial films. Anal. Chem..

[45] Koziara B.T., Akkilic N., Nijmeijer D.C., Benes N.E. (2016). The effects of water on the morphology and the swelling behavior of sulfonated poly(ether ether ketone) films. J. Mater. Sci..

[46] Di Vona M.L., Sgreccia E., Tamilvanan M., Khadhraoui M., Chassigneux C., Knauth P. (2010). High ionic exchange capacity polyphenylsulfone (SPPSU) and polyethersulfone (SPES) cross-linked by annealing treatment: Thermal stability, hydration level and mechanical properties. J. Membr. Sci..

[47] Di Vona M.L., Pasquini L., Narducci R., Pelzer K., Donnadio A., Casciola M., Knauth P. (2013). Cross-linked sulfonated aromatic ionomers via SO2 bridges: Conductivity properties. J. Power Sources.

[48] Knauth P., Sgreccia E., Donnadio A., Casciola M., Di Vona M.L. (2011). Water activity coefficient and proton mobility in hydrated acidic polymers. J. Electrochem. Soc..

[49] Knauth P., Di Vona M. (2012). Sulfonated aromatic ionomers: Analysis of proton conductivity and proton mobility. Solid State Ion..

[50] Mauritz K.A., Moore R.B. (2004). State of understanding of nafion. Chem. Rev..

[51] Gierke T.D., Munn G.E., Wilson F.C. (1981). The morphology in nafion perfluorinated membrane products, as determined by wide- and small-angle x-ray studies. J. Polym. Sci. Polym. Phys. Ed..

[52] Yeager H.L. (1981). Cation and water diffusion in nafion ion exchange membranes: Influence of polymer structure. J. Electrochem. Soc..

[53] Hamann C.H., Hamnett A., Vielstich W. (2007). Electrochemistry.

[54] Jaffrezic-Renault N., Dzyadevych S.V. (2008). Conductometric microbiosensors for environmental monitoring. Sensors.

